# ACKNOWLEDGEMENTS TO REVIEWERS

**DOI:** 10.2174/138920292406231217233357

**Published:** 2023-12-28

Bentham Science Publishers would like to thank and appreciate the co-operation from all reviewers for their constructive comments and feedback on the manuscripts submitted to **Current Genomics**. Their efforts have contributed greatly to the high quality and continuous growth of the journal. Given below is the list of reviewers who reviewed articles for the Journal during 2023:

Münevver Aksoy (Turkey)

Wahid Ali (India)

Daoud Ali (Saudi Arabia)

Arif Nur M. Ansori (Indonesia)

James T. Arnone (USA)

Shuichi Asakawa (Japan)

Shairaz Baksh (Canada)

Matteo Barberis (UK)

Tiago Belintani (Brazil)

Aditi Bhargava (USA)

Pakpoom Boonchuen (Thailand)

Redouane Boulouiz (Morocco)

Rafael D. Cadamuro (Brazil)

Rafael Camacho-Carranza (Mexico)

Marcelo Lattarulo Campos (Brazil)

Mónica Cappetta (Uruguay)

Zeliha Esin Çelik (Turkey)

Chandra P. Chaturvedi (India)

Lin Chen (China)

Ting Jen Rachel Cheng (Taiwan)

Gang Chi (China)

Francesca Luisa Conforti (Italy)

Thiago Magalhães da Silva (Brazil)

Mehdi Dadashpour (Iran)

Kátia Cristina Dantas (Brazil)

Huimin Deng (China)

Kamaludin Dingle (Kuwait)

Leticia Dobler (Brazil)

Kimberly P. Dobrinski (USA)

Yassamine Doubaj (Morocco)

Peiyong Duan (China)

Debajyoti Dutta (India)

Serge J. Edmé (USA)

Abeer Fadda (Qatar)

Yousef Farhaoui (Morocco)

Casey C. Fowler (Canada)

Sara Franzi (Italy)

Alejandra Garcia-Gasca (Mexico)

Fang Ge (China)

Evgin Goceri (Turkey)

Vanessa Gonzalez-Covarrubias (Mexico)

Hongxing Gui (USA)

Mingxiong Guo (China)

Yash Gupta (USA)

Carren Sy Hau (Philippines)

Jose Alberto Hernandez-Eligio (Mexico)

Joerg Hoheisel (Germany)

Guanglong Hu (China)

Lulu Hu (China)

Huateng Huang (China)

Noelle James (USA)

Ruslan N. Kalendar (Kazakhstan)

Assaf Katz (Chile)

Khalid Khfif (Morocco)

Irina V. Khilyas (Russia)

Hyun Min Kim (China)

Mehmet köroğlu (Turkey)

Uttam Kumar (India)

Lela Lackey (USA)

Fagen Li (China)

Haitao Li (China)

Mei Li (China)

Xuming Li (China)

Zhiqing Li (China)

Xiaoping Li (USA)

Chungping Liao (Taiwan)

Thomas Liehr (Germany)

Leonard W. K. Lim (Malaysia)

Wei Sheng Lin (Taiwan)

Jian Ling (China)

Xiangdong Liu (China)

Qian Liu (USA)

Sandra Ludwig (Brazil)

Peixiang Ma (China)

Yiongxin Ma (China)

Rahma Melki (Morocco)

Mohammad Miransari (Iran)

Melisa C. Monteleone (Argentina)

Jianbing Mu (USA)

Pavel V. Nikitin (Russia)

Takayuki Nishimura (Japan)

Olga S. Novikova (USA)

Maria B. Melo De Oliveira (Brazil)

Beata Pająk (Poland)

Raffaele Palmirotta (Italy)

Rui Pang (China)

Marina Perona (Argentina)

Joice F. de Faria Poloni (Brazil)

Lin Qiu (China)

Nallur B. Ramachandra (India)

Donald L. Rockwood (USA)

Diego Romario-Silva (Brazil)

Ana Rossini (Brazil)

Maria P. Rubtsova (Russia)

Ravi Sachidanandam (USA)

Jagajjit Sahu (China)

Richa Salwan (India)

Narendra V. Sankpal (USA)

Winnie W. C. Shum (China)

Prim B. Singh (Russia)

Mahipal Singh Kesawat (India)

Aleksandra V. Sokolova (Russia)

Jeyanthi Suppiah (Malaysia)

Prashanth Suravajhala (India)

Osamu Suzuki (Japan)

Qianyuan Tang (Japan)

Demet Tatar (Turkey)

Iman Tavassoly (USA)

Gestter W. L. Tessarin (Brazil)

Wei Tian (China)

Igor I. Titov (Russia)

Sylvain Tollis (Finland)

Toshifumi Tsukahara (Japan)

Emel Turhan (Turkey)

Asmat Ullah (China)

Hemamalini Vedagiri (India)

Patrick Videau (USA)

Franz Villarroel-Espíndola (Chile)

Virginia K. Walker (Canada)

Lei Wang (China)

Rui Wang (China)

Xingyu Wang (China)

Zhengfeng Wang (China)

Bo Wang (USA)

Richard R.C. Wang (USA)

Jiafa Wu (China)

Guanjue Xiang (USA)

Xiangsheng Xiao (China)

Jiao Yang (China)

Wulin Yang (China)

Stephen Shing Toung Yau (China)

Sibel Yildirim (Turkey)

Zheng Yuan (China) 

Aiming M. M. Zhang (China)

Hao Zhang (China)

Tangjie Zhang (China)

Tao Zhang (China)

Jiangwei Zhu (China)

